# Creation and structure determination of an artificial protein with three complete sequence repeats

**DOI:** 10.1107/S0909049513022164

**Published:** 2013-10-08

**Authors:** Motoyasu Adachi, Rumi Shimizu, Ryota Kuroki, Michael Blaber

**Affiliations:** a Molecular Structural Biology Group, Quantum Beam Science Directorate, Japan Atomic Energy Agency, Shirakatashirane 2-4, Nakagun Tokaimura, Ibaraki 319-1195, Japan; b Department of Biomedical Sciences, Florida State University, Tallahassee, FL 32306-4300, USA

**Keywords:** acidic FGF, Symfoil, protein design

## Abstract

Symfoil-4P is a *de novo* protein exhibiting the threefold symmetrical β-trefoil fold designed based on the human acidic fibroblast growth factor. First three asparagine–glycine sequences of Symfoil-4P are replaced with glutamine–glycine (Symfoil-QG) or serine–glycine (Symfoil-SG) sequences protecting from deamidation, and His-Symfoil-II was prepared by introducing a protease digestion site into Symfoil-QG so that Symfoil-II has three complete repeats after removal of the N-terminal histidine tag. The Symfoil-QG and SG and His-Symfoil-II proteins were expressed in *Eschericha coli* as soluble protein, and purified by nickel affinity chromatography. Symfoil-II was further purified by anion-exchange chromatography after removing the HisTag by proteolysis. Both Symfoil-QG and Symfoil-II were crystallized in 0.1 *M* Tris-HCl buffer (pH 7.0) containing 1.8 *M* ammonium sulfate as precipitant at 293 K; several crystal forms were observed for Symfoil-QG and II. The maximum diffraction of Symfoil-QG and II crystals were 1.5 and 1.1 Å resolution, respectively. The Symfoil-II without histidine tag diffracted better than Symfoil-QG with N-terminal histidine tag. Although the crystal packing of Symfoil-II is slightly different from Symfoil-QG and other crystals of Symfoil derivatives having the N-terminal histidine tag, the refined crystal structure of Symfoil-II showed pseudo-threefold symmetry as expected from other Symfoils. Since the removal of the unstructured N-terminal histidine tag did not affect the threefold structure of Symfoil, the improvement of diffraction quality of Symfoil-II may be caused by molecular characteristics of Symfoil-II such as molecular stability.

## Abstract 2

An artificial protein with three complete sequence repeats was created and the structure was determined by X-ray crystallography. The structure showed threefold symmetry even though there is an amino- and carboxy-terminal. The artificial protein with threefold symmetry may be useful as a scaffold to capture small materials with *C*3 symmetry.

## 1. Introduction

Symmetry is one of the important thema in developing protein structure, function, evolution and design. Although complete structural symmetry is observed in many different natural proteins as homo-oligomerized architectures, structural pseudo­symmetry is also observed in some monomeric proteins. These pseudosymmetric architectures are generally hypothesized as a result of gene duplication and fusion (Sepulveda *et al.*, 1975 ▶; Tang *et al.*, 1978 ▶; McLachlan, 1979 ▶; Inana *et al.*, 1983 ▶). Two distinctly different evolutionary models for the emergence of symmetric protein architecture from a primordial peptide motif have been proposed (Mukhopadhyay, 2000 ▶; Ponting & Russell, 2000 ▶; Liu *et al.*, 2002 ▶; Yadid & Tawfik, 2007 ▶; Akanuma *et al.*, 2010 ▶; Richter *et al.*, 2010 ▶).

In a previous report, we described an experimental top-down symmetric deconstruction (TDSD) of symmetric protein architecture (the β-trefoil fold) using human fibroblast growth factor-1 (FGF-1), a 140 amino acid single-domain globular protein exhibiting characteristic threefold symmetry of the β-trefoil architecture. The TDSD involved sequential introduction of symmetric mutations (targeting core, reverse-turn and β-strand secondary structure, respectively) until a purely threefold symmetric primary structure solution was achieved. Through this approach, we obtained a simplified β-trefoil protein (Symfoil-4P) having a reduced amino acid alphabet size of 16 letters, and enriched in prebiotic amino acids (to 71%) (Lee & Blaber, 2011 ▶; Longo *et al.*, 2013 ▶).

In order to obtain Symfoil with more complete symmetry, and greater chemical stability, we designed a monomeric protein (Symfoil-II) based on the Symfoil-4P protein (Lee & Blaber, 2011 ▶). In the sequence of Symfoil-II, three aspargine–glycine sequences were introduced to improve chemical stabilization from producing charge isomers by deamidation reaction. Furthermore, a protease digestion site was introduced to make the three repeats of Symfoil more complete after removing the N-terminal histidine tag. Here, we report the crystal structure and characteristics of Symfoil-II. Symfoil-II with complete threefold axis may be useful as a scaffold that can capture small *C*3 symmetric compounds using the threefold axis within the Symfoil-II protein.

## 2. Materials and methods

### 2.1. Site-directed mutagenesis

**Figure 1:**
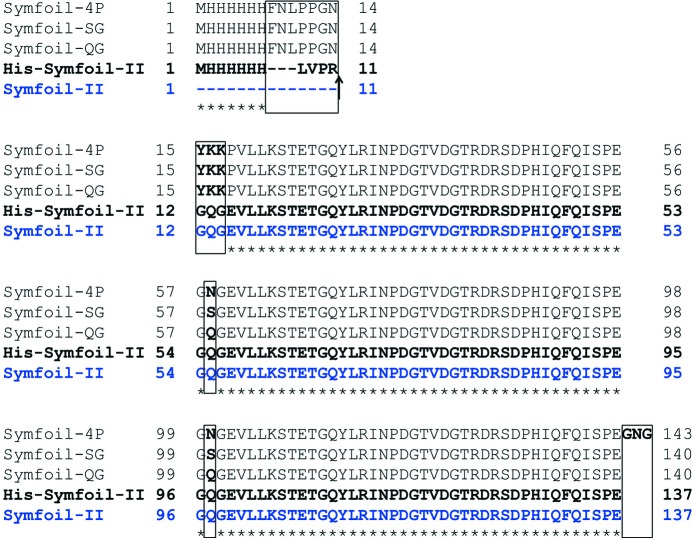
Sequence alignment of Symfoil proteins. Mutated sites are boxed. The arrow indicates the thrombin cleavage site newly introduced in Symfoil-II.

To construct expression plasmids for the mutants, site-directed mutagenesis on the Symfoil-4P in pET-21a vector (Brych *et al.*, 2001 ▶) was achieved by using polymerase chain reaction (PCR). PrimeStar Max DNA polymerase (Takara Bio) was used for the PCR. The PCR products were transfected into *Escherichia coli* HST08 strain without ligation (Takara Bio). Primers used for PCR are listed in Table 1 ▶. For creation of Symfoil-SG and QG, subcloning of the PCR product was repeated three times. In the first reaction, Asn100 was replaced with serine or glutamine. In the secondary reaction, Asn58 was replaced with serine or glutamine. Finally, primers of Cdel_F and Cdel_R were used for deletion of C-terminal three amino acids. The resulting amino acid sequences of Symfoil-QG and Symfoil-SG are shown in Fig. 1 ▶. For preparation of the expression plasmid of His-Symfoil-II, the plasmid template of Symfoil-QG was amplified by using primers N_delQG_F and N_delQG_R as listed in Table 1 ▶. The DNA sequences of the coding region in all plasmids constructed here were confirmed by using ABI Prism 310 DNA sequencer (Applied Biosystems).

### 2.2. Expression and purification

Synthetic polynucleotides coding Symfoil-SG, Symfoil-QG and His-Symfoil-II were expressed using the pET21a(+) plasmid/BL21(DE3) *Escherichia coli* host expression system (Merck). Expression of mutant Symfol proteins followed previously described procedures (Lee & Blaber, 2011 ▶). The cells were resuspended in buffer A [50 m*M* potassium phosphate buffer (pH 7.5) containing 0.1 *M* NaCl] and sonicated. The resultant crude protein solutions were centrifuged at 12000×*g* for 20 min. The obtained supernatants were dialyzed against buffer A, and were applied to a HisTrap FF column (5 ml) (GE-Healthcare) equilibrated by buffer A. The column was washed by buffer A containing 20 m*M* imidazole and eluted by a step gradient of 250 m*M* imidazole. The eluted fractions were dialyzed against buffer A and passed through a HiTrap Heparin HP column (5 ml) (GE-Healthcare) equilibrated by buffer A to remove impurities. The flow-through fractions were collected, and loaded onto a ResourceQ column (3 ml) (GE-Healthcare) equilibrated by buffer A. Elution from the ResourceQ column was achieved by a linear gradient of NaCl.

The histidine tag of His-Symfoil-II was further removed by trypsin (Wako Pure Chemical Industries, Japan), and purified by ResourceQ column chromatography to generate Symfoil-II. Extinction coefficients of E280nm (0.1%, 1 cm) of 3.8, 3.8, 2.9 and 3.1 were used to calculate protein concentrations for the Symfoil-SG, Symfoil-QG, His-Symfoil-II and Symfoil-II, respectively. The final yield was about 15 mg from 1 l of culture.

### 2.3. Gel filtration

To characterize the self-assembly of Symfoil-II, gel filtration chromatography using a Superdex 200 10/300GL column (GE Healthcare) was conducted. The column was equilibrated with 50 m*M* potassium phosphate buffer (pH 7.5) containing 0.2 *M* NaCl. The molecular mass of eluted Symfoil-II was determined by multi-angle laser light scattering (SEC-MALLS). Light-scattering analysis was performed using a miniDAWN detector (Wyatt Technologies).

### 2.4. Crystallization

The purified Symfoil proteins were dialyzed against 50 m*M* sodium phosphate buffer (pH 7.5) containing 100 m*M* NaCl, 10 m*M* ammonium sulfate and 0.5 m*M* EDTA, and then concentrated to 20 mg ml^−1^. Crystallization was performed by hanging-drop vapor diffusion in 0.1 *M* Tris-HCl buffer (pH7.0) containing 1.5–2.0 *M* ammonium sulfate as precipitant. Drops consisting of 2 µl protein solution and 2 µl mother liquor were equilibrated against 1 ml of reservoir solution at 293 K for one week.

### 2.5. Data collection and refinement

Diffraction data of crystals of Symfoil-QG and Symfoil-II were collected using synchrotron radiation sources (λ = 1.00 Å) at beamlines in SPring-8 and KEK, Japan. The crystals were mounted using a nylon cryo-loop (Hampton Research) and were frozen in a liquid-nitrogen stream at 100 K. Diffraction data were collected and indexed, integrated and scaled using the *HKL2000* software package (Otwinowski & Minor, 1997 ▶). A molecular replacement search for non-isomorphous space groups was carried out using the program *Phaser* from the CCP4 suite (McCoy *et al.*, 2007 ▶; Winn *et al.*, 2011 ▶) and coordinates of Symfoil-4P *de novo* designed protein [Protein Data Bank (PDB) code 3o4d] as a search model. Model building and visualization was performed using the *X-tal View* molecular graphics software (McRee, 1992 ▶). The *PHENIX* software package (Zwart *et al.*, 2008 ▶) and the program *REFMAC* (Murshudov *et al.*, 2011 ▶) were used for refinement, in which 5% of the data in the reflection files were set aside for *R*_free_ calculations. The *ARP*/*wARP* automated procedure was used to add solvent molecules (Lamzin & Wilson, 1993 ▶). Atomic models were drawn using the graphics program *Pymol* (DeLano, 2002 ▶).

## 3. Results and discussion

Symfoil-II was designed based on the crystal structure of Symfoil-4P to have more perfect sequence repeats as shown in Fig. 1 ▶. We first removed three NG (Asn–Gly) sequences by changing Asn58 and Asn100 to Ser or Gln and by deleting the Gly141–Asn142–Gly143 sequence to give a Symfoil-SG or Symfoil-QG, respectively, to protect from deamidation during the crystallization experiments. Then, we added a thrombin cut site and GQG sequence to the N-terminal of the first sequence repeat of Symfoil-QG to make His-Symfoil-II. After the removal of the N-terminal histidine tag of His-Symfoil-II, Symfoil-II will be expected to have three complete sequence repeats in one protein as shown in Fig. 1 ▶.

**Figure 2:**
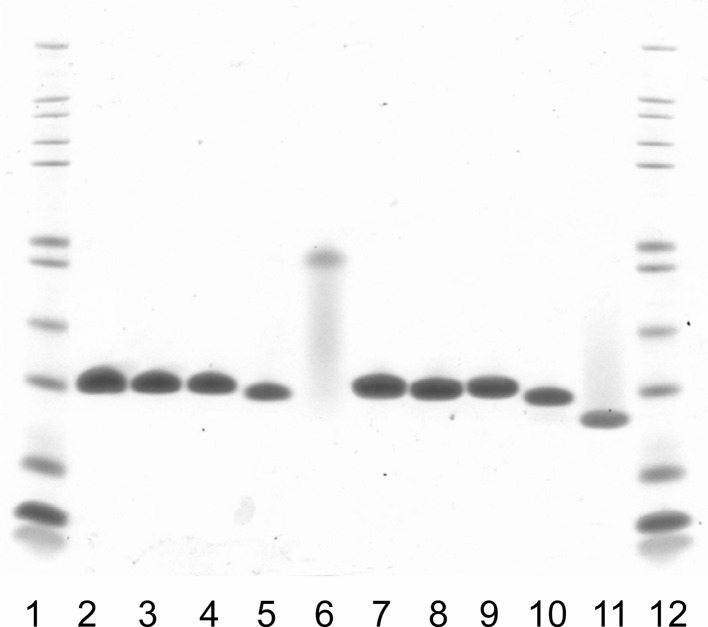
SDS-PAGE analysis of the purified Symfoil proteins. From lanes 2 to 6, the sample is not boiled before loading. From lanes 7 to 11, the sample is boiled before loading. Symfoil-4P: lanes 2 and 7; Symfoil-SG: lanes 3 and 8; Symfoil-QG: lanes 4 and 9; His-Symfoil-II: lanes 5 and 10; Symfoil-II: lanes 6 and 11. Protein size markers of Mark12 (Life Technologies) are shown in lanes 1 and 12.

Symfoil-SG, Symfoil-QG and His-Symfoil-II were prepared after expression using the *E. coli* expression system. The purity of Symfoil-SG, Symfoil-QG and His-Symfoil-II with N-terminal histidine tag and Symfoil-II without N-terminal histidine tag was confirmed by SDS-PAGE (Fig. 2 ▶). The SDS-PAGE showed that the molecular size of His-Symfoil-II (lane 5 and 10) was slightly smaller than those of Symfoil-4P (lane 2 and 7), Symfoil-QG (lane 3 and 8) and Symfoil-SG (lane 4 and 9) because of the removal of the N-terminal YKK sequence. After removal of the histidine tag, Symfoil-II became smaller than other Symfoils as seen in lane 11 with heat treatment. Without heat treatment, Symfoil-II looked extremely large (similar size to its dimer), suggesting that Symfoil-II might form a larger complex. To identify the actual molecular size of Symfoil-II, the molecular size was evaluated by gel filtration equipped with a multi-angle light-scattering detector. The molecular weight of Symfoil-II was, however, estimated to be 14 × 10^3^, which is similar to the theoretical value (13932) for the monomeric Symfoil-II calculated from its primary structure. Although the mechanism for the size shift seen in SDS PAGE is still unclear, stabilization of Symfoil-II against the denaturation by SDS may be a part of the reason why this band shifts. Further assay of the melting experiment is under way.

Now, we obtained Symfoil-II with three complete sequence repeats. We next investigated the effect of the removal of the histidine tag and the Asn–Gly sequence on X-ray diffraction using three independent crystal forms of Symfoil-QG and two independent crystal forms of Symfoil-II. Symfoil-QG crystals diffracted to 2.0, 2.0 and 1.8 Å resolution, respectively, whereas Symfoil-II crystals diffracted to 1.4 and 1.15 Å resolution, respectively, as summarized in Table 2 ▶. Symfoil-II was crystallized into different space groups. The *C*2 space group was uniquely obtained in Symfoil-II and the crystal diffracted to 1.15 Å resolution. The diffraction limit and also the Wilson *B*-factor are shown in Table 2 ▶. This indicates that Symfoil-II diffracted better than the other symfoils with lower *B*-values. These improved diffraction and lower *B*-values in Symfoil-II may be caused by structural stabilization. The close location of the N-terminal and C-terminal in Symfoil-II may give a chance to form an ion pair and the electrostatic stabilization may be part of the reason for its stabilization. Evaluation of the stability of Symfoil-II with and without the histidine tag is the next subject to be investigated.

**Figure 3:**
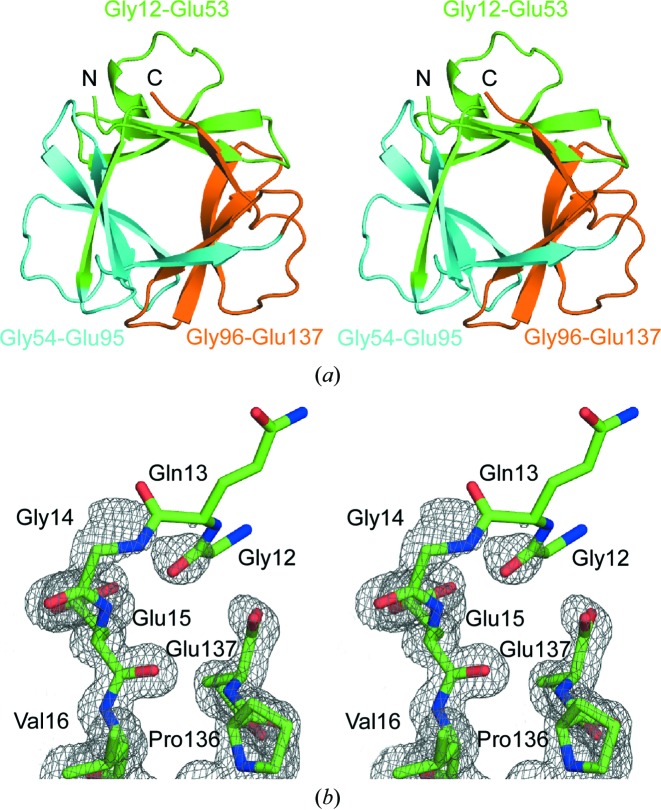
Structure of Symfoil-II in space group *C*2. (*a*) Overall structure of Symfoil-II represented by a ribbon model. The first repeat (residues 12–53 in Fig. 1 ▶) is colored in green, the second repeat (54–95) is colored in cyan and the third repeat (96–137) is colored in orange. (*b*) Structure of N- and C-terminal residues in Symfoil-II. The 2*F*_o_ − *F*_c_ electron density map is contoured at the 1.0σ level.

Crystal structures of the Symfoil-QG and Symfoil-II were determined and the refinement statistics are summarized in Table 2 ▶. The overall structure of Symfoil-II is shown in Fig. 3(*a*) ▶. The RMS difference between the structures of Symfoil-QG and Symfoil-II in the *I*222 crystal form is 0.39 Å, indicating that the structural difference caused by the removal of the N-terminal sequence is quite small. Location of the N-terminal histidine tag of Symfoil-QG was not determined in any crystals obtained in this study. Electron densities for the loop region connecting three repeats in Symfoil-QG and Symfoil-II were still invisible, but became clearer in the structure of Symfoil-II determined to 1.15 Å resolution (Fig. 3*b* ▶). Assuming that Symfoil-II with complete three sequence repeats has an ion pair at the N- and C-terminal, the structure is almost perfect threefold symmetry. RMS positional differences after application of the rotation matrix calculated using the structures of each repeat of Symfoil-QG and Symfoil-II were less than 0.37 Å for Symfoil-QG and less than 0.37 Å for Symfoil-II, indicating that both Symfoils have threefold symmetry including the shape of the central cavity.

In conclusion, we succeeded in preparation of artificial protein having three complete sequence repeats. Prepared Symfoil-II resulted in improving the X-ray diffraction and the structural details were figured out. We are now attempting to convert the ion pair of the N- and C-terminal with an amide bond to prepare a circular Symfoil, which may be useful as a scaffold to capture molecules having *C*3 symmetry (Gibson & Castaldi, 2006 ▶) by virtue of specific interaction with the threefold axes of symmetry present in Symfoil-II.

## Figures and Tables

**Table 1:** Primers used for site-directed mutagenesis

Mutation	Name of primer	Sequence
N58S	SG_Site1_F	AAGGCAGTGGTGAAGTTCTG
	SG_Site1_R	CTTCACCACTGCCTTCCGGG
N58Q	QG_Site1_F	CGGAAGGCCAGGGTGAAGTTCTG
	QG_Site1_R	CACCCTGGCCTTCCGGGGAG
N100S	SG_Site2_F	AGGGTAGCGGCGAGGTACTC
	SG_Site2_R	CCTCGCCGCTACCCTCAGGG
N100Q	QG_Site2_F	CTGAGGGTCAGGGCGAGGTACTC
	QG_Site2_R	CGCCCTGACCCTCAGGGGAA
Deletion	Cdel_F	GTCGACAAGCTTGCGGCCGCACTCGAGCACCACCACCACCACCACTGA
	Cdel_R	CGCAAGCTTGTCGACTTAGCCCTGTCACTCTGGGCTAATCTGGAAT
Symfoil-II	N_delQG_F	CCGCGCGGTCAAGGTGAAGTGCT­TCTTAAGAGCACTGAAACCGG­CCAG
	N_delQG_R	ACCTTGACCGCGCGGCACCAGATG­GTGATGGTGATGGTGCATATGTATATC

**Table 2:** X-ray data collection and refinement statistics for Symfoil molecules Values in parentheses are for the highest-resolution shell.

	Symfoil-QG	Symfoil-QG	Symfoil-QG	Symfoil-II	Symfoil-II
Data collection					
Beamline	SPring-8 BL38B1	PF BL17A	SPring-8 BL38B1	PF BL5A	PF BL5A
Space group	*I*222	*C*222_1_	*R*3	*I*222	*C*2
Unit-cell parameters (Å, °)	*a* = 50.4, *b* = 53.0, *c* = 84.8	*a* = 58.3, *b* = 66.5, *c* = 66.6	*a* = 55.2†, *b* = 55.2†, *c* = 125.6†	*a* = 51.1, *b* = 53.2, *c* = 84.4	*a* = 81.4, *b* = 47.9, *c* = 57.2 β = 133
Resolution (outer shell) (Å)	27.7–2.00 (2.07–2.00)	26.7–1.80 (1.86–1.80)	26.2–2.00 (2.07–2.00)	25.6–1.40 (1.45–1.40)	41.8–1.15 (1.19–1.15)
No. of observed reflections	48805	60109	42312	145900	235712
No. of unique reflections	7608 (605)	11849 (1124)	8819 (913)	22274 (2133)	55243 (5300)
Redundancy	6.4 (5.8)	5.1 (4.6)	4.8 (4.3)	6.6 (3.8)	4.3 (3.8)
Completeness (%)	95.4 (78.4)	96.2 (92.1)	91.6 (95.8)	96.3 (93.7)	96.0 (93.2)
*I*/σ(*I*)	25.0 (2.8)	24.9 (3.3)	14.5 (2.6)	42.5 (2.0)	43.1 (3.0)
*R*_merge_	0.109 (0.503)	0.093 (0.415)	0.134 (0.433)	0.067 (0.601)	0.049 (0.649)
Wilson plot *B*-factor (Å^2^)	32.4	20.4	23.2	18.2	13.7

Refinement statistics					
Resolution (Å)	27.7–2.00	26.7–1.80	26.3–2.00	25.6–1.40	41.8–1.05
No. of water molecules	68	82	127	113	199
*R* factor/*R*_free_	0.212/0.305	0.190/0.270	0.221/0.307	0.230/0.300	0.146/0.176
R.m.s.d. bonds (Å)	0.014	0.018	0.014	0.021	0.030
R.m.s.d. angles (°)	1.693	2.025	1.642	2.424	2.539
Program	*REFMAC*	*REFMAC*	*REFMAC*	*REFMAC*	*PHENIX/REFMAC*

† Hexagonal obverse setting.

## References

[bb1] Akanuma, S., Matsuba, T., Ueno, E., Umeda, N. & Yamagishi, A. (2010). *J. Biochem.* **147**, 371–379. 10.1093/jb/mvp179 19889751

[bb2] Brych, S. R., Blaber, S. I., Logan, T. M. & Blaber, M. (2001). *Protein Sci.* **10**, 2587–2599. 10.1110/ps.ps.34701 PMC2374030 11714927

[bb3] DeLano, W. L. (2002). *PyMOL*, http://www.pymol.org. 10.1016/s0959-440x(02)00283-x 11839484

[bb4] Gibson, S. E. & Castaldi, M. P. (2006). *Chem. Commun.* pp. 3045–3062. 10.1039/b602237e 16855685

[bb5] Inana, G., Piatigorsky, J., Norman, B., Slingsby, C. & Blundell, T. (1983). *Nature (London)*, **302**, 310–315. 10.1038/302310a0 6835368

[bb6] Lamzin, V. S. & Wilson, K. S. (1993). *Acta Cryst.* D**49**, 129–147. 10.1107/S0907444992008886 15299554

[bb7] Lee, J. & Blaber, M. (2011). *Proc. Natl Acad. Sci. USA*, **108**, 126–130. 10.1073/pnas.1015032108 PMC3017207 21173271

[bb8] Liu, L., Iwata, K., Yohda, M. & Miki, K. (2002). *FEBS Lett.* **528**, 114–118. 10.1016/s0014-5793(02)03264-7 12297289

[bb9] Longo, L. M., Lee, J. & Blaber, M. (2013). *Proc. Natl Acad. Sci. USA*, **110**, 2135–2139. 10.1073/pnas.1219530110 PMC3568330 23341608

[bb10] McCoy, A. J., Grosse-Kunstleve, R. W., Adams, P. D., Winn, M. D., Storoni, L. C. & Read, R. J. (2007). *J. Appl. Cryst.* **40**, 658–674. 10.1107/S0021889807021206 PMC2483472 19461840

[bb11] McLachlan, A. D. (1979). *J. Mol. Biol.* **133**, 557–563. 10.1016/0022-2836(79)90408-x 537058

[bb12] McRee, D. (1992). *J. Mol. Graph.* **10**, 44–46.

[bb13] Mukhopadhyay, D. (2000). *J. Mol. Evol.* **50**, 214–223. 10.1007/s002399910024 10754063

[bb14] Murshudov, G. N., Skubák, P., Lebedev, A. A., Pannu, N. S., Steiner, R. A., Nicholls, R. A., Winn, M. D., Long, F. & Vagin, A. A. (2011). *Acta Cryst.* D**67**, 355–367. 10.1107/S0907444911001314 PMC3069751 21460454

[bb15] Otwinowski, Z. & Minor, W. (1997). *Methods Enzymol.* **276**, 307–326. 10.1016/S0076-6879(97)76066-X 27754618

[bb16] Ponting, C. P. & Russell, R. B. (2000). *J. Mol. Biol.* **302**, 1041–1047. 10.1006/jmbi.2000.4087 11183773

[bb17] Richter, M., Bosnali, M., Carstensen, L., Seitz, T., Durchschlag, H., Blanquart, S., Merkl, R. & Sterner, R. (2010). *J. Mol. Biol.* **398**, 763–773. 10.1016/j.jmb.2010.03.057 20363228

[bb18] Sepulveda, P., Marciniszyn, J., Liu, D. & Tang, J. (1975). *J. Biol. Chem.* **250**, 5082–5088. 1097438

[bb19] Tang, J., James, M. N., Hsu, I. N., Jenkins, J. A. & Blundell, T. L. (1978). *Nature (London)*, **271**, 618–621. 10.1038/271618a0 24179

[bb20] Winn, M. D., Ballard, C. C., Cowtan, K. D., Dodson, E. J., Emsley, P., Evans, P. R., Keegan, R. M., Krissinel, E. B., Leslie, A. G. W., McCoy, A., McNicholas, S. J., Murshudov, G. N., Pannu, N. S., Potterton, E. A., Powell, H. R., Read, R. J., Vagin, A. & Wilson, K. S. (2011). *Acta Cryst.* D**67**, 235–242. 10.1107/S0907444910045749 PMC3069738 21460441

[bb21] Yadid, I. & Tawfik, D. S. (2007). *J. Mol. Biol.* **365**, 10–17. 10.1016/j.jmb.2006.09.055 17054983

[bb22] Zwart, P. H., Afonine, P. V., Grosse-Kunstleve, R. W., Hung, L. W., Ioerger, T. R., McCoy, A. J., McKee, E., Moriarty, N. W., Read, R. J., Sacchettini, J. C., Sauter, N. K., Storoni, L. C., Terwilliger, T. C. & Adams, P. D. (2008). *Methods Mol. Biol.* **426**, 419–435. 10.1007/978-1-60327-058-8_28 18542881

